# Impacts of Different Light Spectra on CBD, CBDA and Terpene Concentrations in Relation to the Flower Positions of Different *Cannabis Sativa* L. Strains

**DOI:** 10.3390/plants11202695

**Published:** 2022-10-13

**Authors:** Philipp Reichel, Sebastian Munz, Jens Hartung, Stiina Kotiranta, Simone Graeff-Hönninger

**Affiliations:** 1 Agronomy, Institute of Crop Science, University of Hohenheim, 70599 Stuttgart, Germany; 2 Biostatistics, Institute of Crop Science, University of Hohenheim, 70599 Stuttgart, Germany; 3 Department of Agricultural Sciences, Viikki Plant Science Centre, University of Helsinki, P.O. Box 27, FI-00014 Helsinki, Finland

**Keywords:** light, cannabis, LED, secondary metabolites

## Abstract

Cannabis is one of the oldest cultivated plants, but plant breeding and cultivation are restricted by country-specific regulations. The plant has gained interest due to its medically important secondary metabolites, cannabinoids and terpenes. Besides biotic and abiotic stress factors, secondary metabolism can be manipulated by changing light quality and intensity. In this study, three morphologically different cannabis strains were grown in a greenhouse experiment under three different light spectra with three real light repetitions. The chosen light sources were as follows: a CHD Agro 400 ceramic metal-halide lamp with a sun-like broad spectrum and an R:FR ratio of 2.8, and two LED lamps, a Solray (SOL) and an AP67, with R:FR ratios of 13.49 and 4, respectively. The results of the study indicated that the considered light spectra significantly influenced CBDA and terpene concentrations in the plants. In addition to the different light spectra, the distributions of secondary metabolites were influenced by flower positions. The distributions varied between strains and indicated interactions between morphology and the chosen light spectra. Thus, the results demonstrate that secondary metabolism can be artificially manipulated by the choice of light spectrum, illuminant and intensity. Furthermore, the data imply that, besides the cannabis strain selected, flower position can have an impact on the medicinal potencies and concentrations of secondary metabolites.

## 1. Introduction

Around the plant *Cannabis sativa* L., a burgeoning industry has developed due to the changing regulatory regimes in Europe towards legalization. Additionally, the plant is in the spotlight of various research projects around the world, which is also evident from the increase in the number of publications in recent years [1]. At the centre of interest is the unique secondary metabolism of cannabis, which is also responsible for its medical purposes.

Recently, important steps have been taken in furthering the understanding of terpene synthesis [2] and the underlying genetic framework [3,4]. In addition, the impacts of growing conditions, e.g., lighting [5], fertilization [6,7] and pruning techniques [8,9], on cannabinoid and terpene synthesis, which define the medical potential of cannabis, were examined.

**Figure 1:**
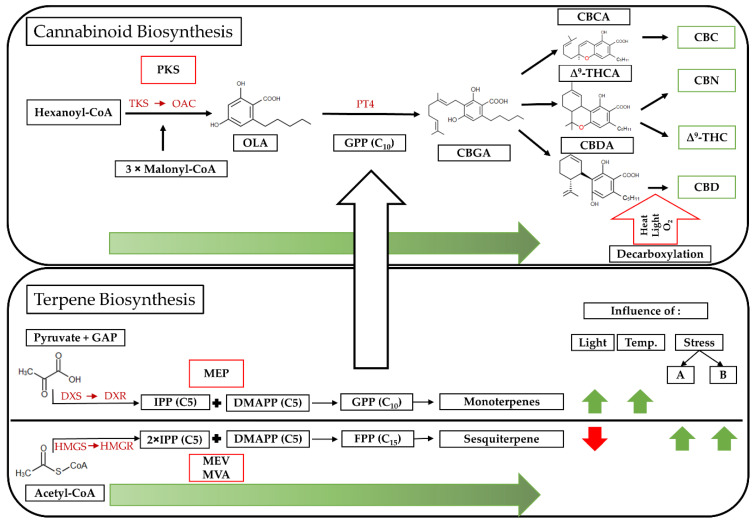
Simplified Cannabis and Terpene Biosynthesis based on [1,3,11,24,25]. Pathways are outlined in red, and important enzymes are highlighted in red. CBCA: Cannabichromenic acid; CBC: Cannabichromene; Δ^9^-THCA: Δ^9^-Tetrahydrocannabinolic acid; Δ^9^-THC: Δ^9^-Tetrahydrocannabinol; CBN: Cannabinol; CBDA: Cannabidiolic acid; CBD: Cannabidiol.

Secondary metabolites of *Cannabis sativa* L. accumulate mainly in the trichomes of the female flowers and in the leaves surrounding the inflorescences, known as sugar leaves. The formation of these compounds is preceded by a cascade of metabolic processes that can be divided into three different pathways: (1) the polyketide synthase (PKS), (2) the mevalonic acid (MVA)–cytosolic mevalonate (MEV) and (3) the plastidial methylerythritol pathway (MEP). PKS produces olivetolic acid (OLA) [10], which is the starting compound for cannabinoid synthesis. Two synthesis pathways are responsible for terpenes: the MVA [11] or MEV [12] pathway and the MEP pathway [13], each of which has dimethylallyl diphosphate (DMAPP) as the end product [14]. In the MEV/MVA pathway, DMAPP is further condensed to farnesyl pyrophosphate (FPP), which forms the precursor for sesquiterpenes [15]. On the other hand, MEP synthesizes geranyl diphosphate (GPP) from DMAPP and is responsible for the formation of monoterpenes. Additionally, GPP together with OLA forms the precursor of all cannabinoids, cannabigerolic acid (CBGA) [16]. Hence, GPP is the connection between monoterpene and cannabinoid biosynthesis. Each pathway is determined by key enzyme steps. During cannabinoid synthesis, tetraketide synthase (TKS) plays an important role, followed by olivetolic acid cyclase (OAC) [17]. Of central importance is geranylpyrophosphate:olivetolate geranyltransferase (PT4) for the formation of CBGA. Terpene biosynthesis involves distinct enzyme sequences for the formation of mono- and sesquiterpenes. For monoterpenes, 1-deoxy-D-xylulose 5-phosphate synthase (DXS) and 1-deoxy-D-xylulose 5-phosphate reductoisomerase (DXR) are crucial, initial enzymes. Hydroxymethylglutaryl-CoA synthase (HMGS) followed by hydroxymethylglutaryl-CoA reductase (HMGR), in contrast, are involved in the formation of sesquiterpenes [18] (Figure 1).

Every setting in the cultivation system of *Cannabis sativa* L. is accompanied by the up- and down-regulation of enzyme expressions, which leads to increase or decrease in the respective end products. Research on the influence of the enzyme steps in cannabinoid biosynthesis is still in its infancy. For terpene biosynthesis, however, knowledge can be derived from other plant species. Major influences are light, temperature and abiotic and biotic stresses, which affect the flow direction of isoprenoid precursors between the MEP and MEV/MVA pathways [18], through modulating enzyme expression. Increasing light intensity leads to a decrease in the MEV/MVA pathway, whereas abiotic and biotic stresses can enhance it [19,20,21]. By contrast, the MEP pathway is promoted by light intensity and temperature [22,23] (Figure 1).

For the final conversion into the respective mono- and sesquiterpenes, terpene synthases (TPSs) [25,26,27] are responsible, which can be classified into different clustering gene groups [3,4] and may be light-mediated [28]. According to [4], TPS-a (caryophyllene and humulene), TPS-g (linalool and nerolidol) and TPS-b (β-Myrcene, limonene, α-pinene and monoterpenes in general) are responsible for the respective terpene syntheses. Despite the progress in deciphering the genetic principles of TPSs, actual terpene profiles are still difficult to predict [3]. This is particularly due to the fact that TPSs are dependent on external factors which modulate enzyme activity, such as light [5], temperature [29], nutrition [6] and other abiotic and biotic stresses [30].

In addition, the developmental stage of the plant has a further effect on the distribution and composition of terpenes and cannabinoids [31]. In general, terpene concentrations are highest in the reproductive plant parts [32], peak before and during flower maturity [33] and can vary within a plant [34]. This is primarily because terpene turnover depends on the photosynthesis potential, which is subject to variation due to light intensity being reduced by the shading of plant organs [35]. The utilization of photosynthates [33] also plays an active role in secondary metabolism, especially in the case of *Cannabis sativa* L., as the sugar leaves surrounding the inflorescence serve as strong sinks in the late flowering phase [36]. Therefore, it can be hypothesized that the shading of flowers, and therefore also of the surrounding sugar leaves, leads to heterogeneous concentrations of secondary metabolites in flowers, depending on the position of the buds on the plant.

Several reviews regarding *Cannabis sativa* L. and the influence of light on yield and secondary metabolism have been published [1,24,37]. Based on the published information, looking at current cannabis light research, ceramic metal-halide lamps (CMHs), high-pressure sodium lamps (HPSs) and light-emitting diodes (LEDs) are established lighting sources used in the cannabis industry. In the context of light, the action spectrum [38] in the range of 400–700 nm is of fundamental importance, as it defines the photosynthetically active radiation (PAR) and includes all wavelengths, except FR (>700 nm). Considering the spectral distributions in recent publications, it is noticeable that mainly full-spectrum lights, which trigger multiple photoreceptors, have been used [39,40]. Nevertheless, some key messages have already been established, such as an increase in visible light, which includes the blue, green and red spectra, which are considered to increase cannabinoids [41,42]. However, a statement on the influence of light spectra on terpene synthesis in *Cannabis sativa* L. is currently inconclusive [24].

In this study, we focus on the major differences in the lights used, namely, the R:FR ratios and the associated activation of the photoreceptors Pr and Pfr [43]. Of particular interest in this context are the phytochromes PHYA, PHYB and PHYC [44], most notably PHYB [35], which seems to play a central role in shade avoidance and may even influence flowering [45]. Shade avoidance, caused by a lowered R:FR ratio in a spectrum, leads to significant morphological changes in *Cannabis sativa* L. [36], and we assume that it also plays an important role in secondary metabolism. The conformations of phytochromes can be influenced by R:FR ratios; low ratios shift the equilibrium towards the inactive Pr form, whereas higher ratios shift it towards Pfr [46]. In *Arabidopsis thaliana*, Pfr has already been shown to enhance terpene synthesis by affecting the MEP pathway through the degradation of phytochrome-interacting factors (Pifs) [47].

We hypothesize that: (I) each strain is characterized by various expressions of *Cannabis sativa* L. (Cs) TPSs due to its genetic heritage, which results in the formation of genetically diverse terpene profiles, present in all flower positions; (II) due to the grouping of the CsTPSs into gene groups, different terpenes are connected and correlate with each other; (III) due to the morphologically induced shading of flowers and the associated low photosynthesis rates (PRs), different concentrations occur in the respective flower positions, with the highest concentration in the constant, fully illuminated main top bud (MTB); (IV) light spectra with a high R:Fr ratio can lead to activation of the phytochrome Pfr, which positively influences the enzyme expression of the MEP pathway by down-regulation of Pifs. This is finally reflected in increased monoterpene and CBDA concentrations in the MTB.

## 2. Materials and Methods

A detailed description of the experimental setup, plant materials, growing conditions and destructive samplings can be found in [36]. In the following, only the most important information is given.

### 2.1. Experimental Setup

**Figure 2:**
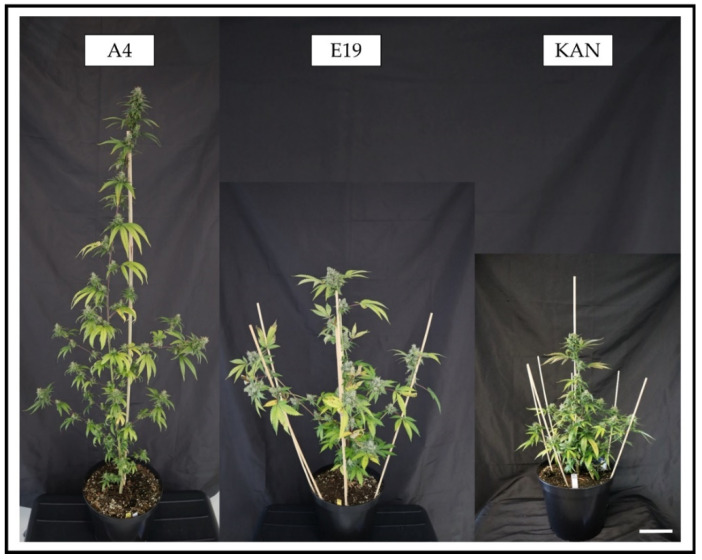
Exemplary plants of the strains A4, E19 and KAN at final harvest, grown under the SOL light spectra (White bar at bottom right = 10 cm).

A greenhouse experiment was carried out at the University of Hohenheim (Stuttgart, Germany) between 10 December and 28 March 2021. The phytocannabinoid-rich *Cannabis sativa* L. strains A4, Kanada (AI FAME, Wald-Schönengrund, Switzerland) (KAN) and E19 (Super Strains, Bladel, The Netherlands) (Figure 2) were grown under three light sources: two LEDs, namely, a Solray385^®^ (SOL) and an AP67 (both obtained from Valoya Oy, Helsinki, Finland), and one ceramic metal-halide lamp, a CHD Agro 400 (DH Licht GmbH, Wülfrath, Germany). Each light source was replicated three times. Light sources were randomized to tables according to a randomized complete block design. The plants of the three strains were randomized according to a row–column design (3 × 4) within each table and thus to each light-source-by-replicate combination. Thus, there were four plants for each strain in each light-source-by-replicate combination. Plants were harvested at three harvest times, with two remaining plants harvested at the final harvest. The design has similarities to a split-plot design, in the sense that light sources as the main-plot factors and strains-by-harvest as sub-plot factors were randomized to different randomization units. The design varies from a split-plot design, however, as there were two plants harvested at the final harvest for each strain-by-main-plot combination. Throughout the experiment, the night temperature was 18 °C and mean day temperatures showed minor variations depending on the light source, with 23.5 °C under AP67, 22.4 °C under SOL and 22.8 °C under CHD, respectively. Humidity varied from 40.5 to 80.1%, and the CO_2_ concentration was between 390 and 450 ppm. Water was provided as needed, according to horticultural standards.

### 2.2. Plant Materials and Growing Conditions

The experimental plants were propagated from their respective mother plants. The plants were repotted in Substrate 5 (Klasmann-Deilmann, Geeste, Germany) three times during their growth. After a rooting period of 21 days, rooted cuttings were transferred to round pots (9 cm), after 18 days to 14 cm pots and after 28 days to the final pot size of 29 cm (Lamprecht-Verpackungen GmbH, Göttingen, Germany), during the transition from long to short days. The fertilization schedule was constant for all treatments and included two different fertilizers. Plantaactiv 18-12-18 Type A was used during the long-day and Plantaactive 10-20-30 Type B (Hauert, Grossaffoltern, Switzerland) during the short-day period. Over 12 weeks, in total, 659.1 mg of N, 659.4 mg of P and 989,1 mg of K were applied per plant.

### 2.3. Data Collection

#### 2.3.1. Destructive Sampling

Plants were destructively sampled three times during the experiment. The first two harvests included 27 plants each, the final harvest the remaining 54 plants, with a total number of 108 experimental plants. For the current study, data from the final harvest were used. The final harvest date was determined when 70% of the pistils had darkened [48]. Harvest date varied depending on the strain: A4 was harvested after 7 weeks (80 DAP), KAN in the middle of week 8 (85 DAP) and E19 at the end of week 8 (88 DAP) of the short-day period. Harvesting was carried out in bundles, according to light spectra. The flower yield of each plant was divided into the main top bud (MTB), side top buds (STBs) and the remaining flowers (SBs).

#### 2.3.2. Photosynthetic Rate

The photosynthetic rate was measured with a GFS-3000 (Heinz Walz GmbH, Effeltrich, Germany). To record photosynthetic rates (A), the conditions in the measurement chamber of the device were set to 30 °C leaf temperature, 50% humidity and an ambient CO_2_ concentration between 399 and 410 ppm. The red:far-red ratios were simulated in LED chambers, according to [49]. The KAN plants used for this purpose, grown under controlled greenhouse conditions, were in the fifth week of flowering (56 DAP), and the first fully developed main leaf (rank 4) was used.

### 2.4. Cannabinoid and Terpene Analysis

#### 2.4.1. Terpene Analysis

The terpene analysis method from [50] was implemented. In the following, only the most important information is given.

Per sample, two Agilent 20 mL headspace vials were filled with 50 mg dried and ground material. The samples were analyzed using an Agilent 8860 GC System with the following components: an Agilent 7697A Headspace Sample, an Agilent 5977B GC/MSD (Residual Solvent Analyzer) and an Agilent VF-35 column (30 m × 0.25 mm, 0.25 μm). The temperature gradient program used to separate mono- and sesquiterpenoids was as follows: vial equilibration consisted of 10 min at 60 °C, then a ramp of 45 °C/min up to 150 °C, with 0 min hold, followed by a ramp of 35 °C/min up to 250 °C, with 0.5 min hold. The run time was 16 min. The maximum injector temperature was 260 °C. The split ratio was 100:1. The carrier gas (helium) column flow rate was 3.0 mL/min. The MSD source temperature was set to 300 °C, the quadrupole temperature was 150 °C and the transfer line temperature was 260 °C. For quantitation, a seven-point calibration curve from 10 ppm to 1250 ppm was created, consisting of cannabis terpene standard #1 (Restek, Bellefonte, PA, USA). The calibration levels used were: 0, 20, 50, 100, 200, 500 and 1250 ppm. Quantifier and qualifier ions for each compound were taken from [11].

#### 2.4.2. Cannabinoid Analysis

The air-dried flower materials were analyzed by high-pressure liquid chromatography (1290 Infinity II LC System, Agilent, Santa Clara, CA, USA), according to [51]. Two vials per sample, with 100 ± 10 mg of milled sample, were dissolved in 25 mL of a 90% methanol–10% chloroform (*v/v*) (9 + 1) composite and extracted in an ultrasonic bath for 30 min. The extract was filtered through polytetrafluorethylene (PTFE) syringe filters, 0.45 μm (Macherey-Nagel GmbH & Co. KG, Dueren, Germany), into an HPLC vial and injected into the HPLC system. Cannabinoids were quantified at a detection wavelength of 230 nm. External calibration of cannabinoid quantification was performed, using two standards (CAN1 and CAN2) containing the target compounds (CAN1: CBD 2%, (Lipomed, Arlesheim, Switzerland), CBDA 10%; and CAN2: CBG 2%, CBGA 2% (Echo Pharmaceuticals BV, Weesp, The Netherlands)).

### 2.5. Statistical Analysis

Data were analysed using the following model:

(1) $$y_{hjklmnp}=\mu +b_{h}+t_{hj}+r_{hjm}+c_{hjn}+p_{hjmn}+\tau_{k}+\varphi_{j}+\rho_{l}+{\left(\tau \varphi \right)}_{kj}+{\left(\tau \rho \right)}_{kl}+{\left(\varphi \rho \right)}_{jl}+{\left(\tau \varphi \rho \right)}_{kjl}+e_{hjklmnp}$$

where $y_{hjklmnp}$ is the observation of the plant *p* located at the *m*-th row, the *n*-th column, on the *j*-th table of the -*h*th room treated with the *k*-th strain, the *j*-th light spectra and the *l*-th flower position; μ is the intercept; $b_{h}$, $t_{hj}$, $r_{hjm}$, $c_{hjn}$ and $p_{hjmn}$ are the random block effects of the *h*-th room, the *j*-th table, the *m*-th row on the *j*-th table, the *n*-th column on the *j*-th table, and the plant grown in row *m*, column *n* of table *j* in room *h*, respectively; $\tau_{k}$, $\varphi_{j}$ and $\rho_{l}$ are the fixed effects of the *k*th strain, the *j*-th light spectra and the *l-*th flower position; ${\left(\tau \varphi \right)}_{kj}$, ${\left(\tau \rho \right)}_{kl}$, ${\left(\varphi \rho \right)}_{jl}$ and ${\left(\tau \varphi \rho \right)}_{kjl}$ are the fixed two- and three-way interaction effects between the corresponding factors involved; and $e_{hjklmnp}$ is the error of $y_{hjklmnp}$. Error effects were allowed to have heterogeneous variances if this increased model fitted according to the AIC. As the design was similar to a split-plot design, $t_{hj}$ served as the main-plot error. Normal distributions and homogeneous variances of residuals were checked graphically via residual plots. Means were compared using Tukey’s test and were displayed using a letter display [52].

Additionally, strain-by-spectra-by-position means were calculated for all 12 traits. These simple means were standardized to have a mean of zero and a variance of one. A principal component analysis (PCA) was applied on these standardized means. The two first components were presented in a biplot, using the default setting of the biplot macro for SAS (factype = SYM).

## 3. Results

A detailed description of the results concerning yield, morphology and growth trajectories can be found in [36].

The concentrations of secondary metabolites had to be interpreted in relation to the total dry flower yield to consider a potential dilution effect. The strain E19 achieved a significantly higher yield of 15.69 g DW plant^−1^ compared to KAN (10.72 g DW plant^−1^) and A4 (6.15 g DW plant^−1^). Similarly, under the AP67 (13.23 g DW plant^−1^) and SOL (10.95 g DW plant^−1^) LED spectra, a significant increase in yield was observed compared to CHD (8.38 g DW plant^−1^).

### 3.1. CBD and CBDA

**Figure 3:**
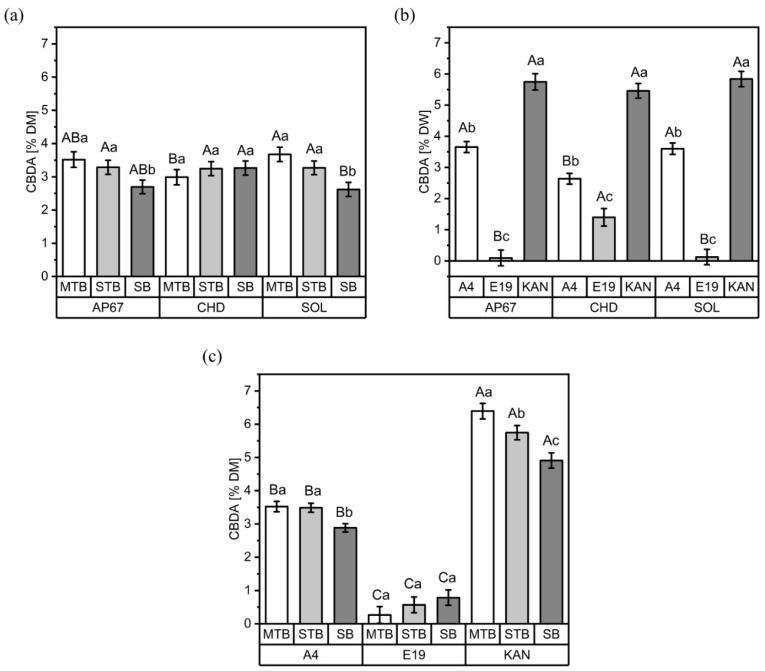
CBDA concentrations (% DW) for the three flower positions (MTB: main top bud, SBs: side buds, STBs: side top buds) under the three light spectra (SOL, AP67 and CHD) (**a**), for the three strains (A4, E19 and KAN) under the three light spectra (**b**) and for the three flower positions of the respective strains (**c**). Means covered by at least one identical letter did not differ significantly, as indicated by a Tukey test (α = 0.05). Lowercase letters indicate comparisons between flower positions (**a**) or strains (**b**) within light spectra, or flower positions within strains (**c**); uppercase letters indicate comparisons between light spectra within flower positions (**a**) or strains (**b**), or strains within flower positions (**c**). Error bars indicate the standard errors of the means (*n* = 3).

No differences in CBD concentrations between the light treatments were observed. The average CBD concentrations were 0.61%, 0.55% and 0.45% for Kanada, A4 and E19, respectively. In contrast, the CBDA concentrations displayed significant differences in the interactions between light spectra and flower positions, as well as between light spectra and strains and individual flower positions within the respective strains (Figure 3).

CBDA concentrations varied according to flower position, especially under the LED treatments, with higher concentrations measured for the main (MTBs) and side top buds (STBs) and lower concentrations for the side buds (SBs). Such variation in CBDA concentration in the CHD plants was not observed (Figure 3a). The effects of the light spectra on CBDA concentrations were not consistent between the cultivars (Figure 3b); no difference in CBDA concentration was observed in the cultivar Kanada; however, a 38% increase was detected in cultivar A4 under the LED treatments compared to CHD, and, on the contrary, the CBDA concentration for E19 was 11-fold higher under CHD compared to the LED treatments.

Across the three light spectra, each strain exhibited different quantities and distributions of CBDA concentrations at different flower positions (Figure 3c). Kanada had the highest CBDA concentration (6.39%), followed by A4 (3.52%) and E19 (0.78%). Furthermore, Kanada showed a significant gradient across flower positions, following the order MTBs > STBs > SBs. The lowest CBDA concentration for A4 was found in the SB flower position; however, MTBs and STBs showed similar concentrations. No significant differences were found for strain E19 with respect to flower positions.

### 3.2. Terpenes

**Figure 4:**
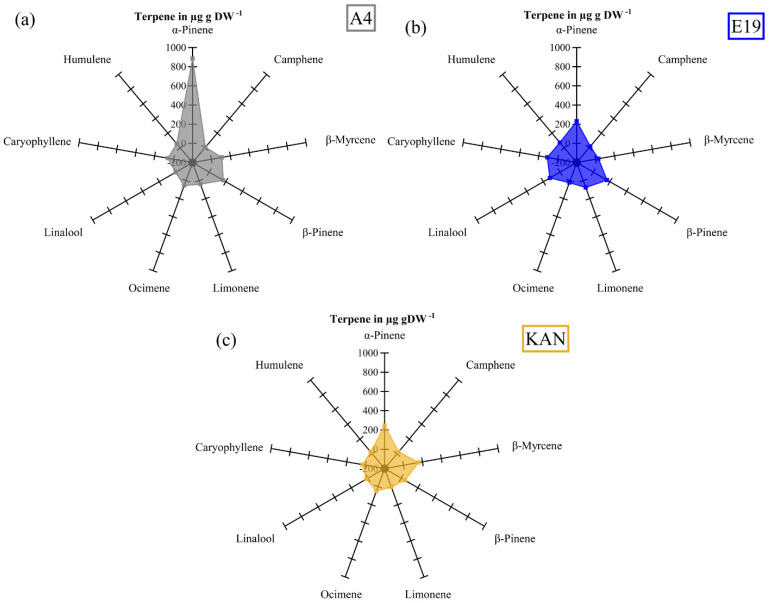
Radar chart of the terpene profile for the three strains: A4 (**a**), E19 (**b**) and KAN (**c**).

The terpene profiles showed clear differences between strains, with A4 being dominated by α-pinene and additional higher concentrations of β-pinene and β-myrcene (Figure 4a), whereas E19 was characterized by linalool, caryophyllene and β-pinene (Figure 4b). The strain KAN showed a more homogeneous distribution, with higher concentrations of ocimene and β-myrcene (Figure 4c).

Additionally, the terpenes exhibited significant two-way interactions between light, strain and flower position. The results are therefore presented separately for the different interactions.

#### 3.2.1. Interactions between Light Spectra and Strains

**Figure 5:**
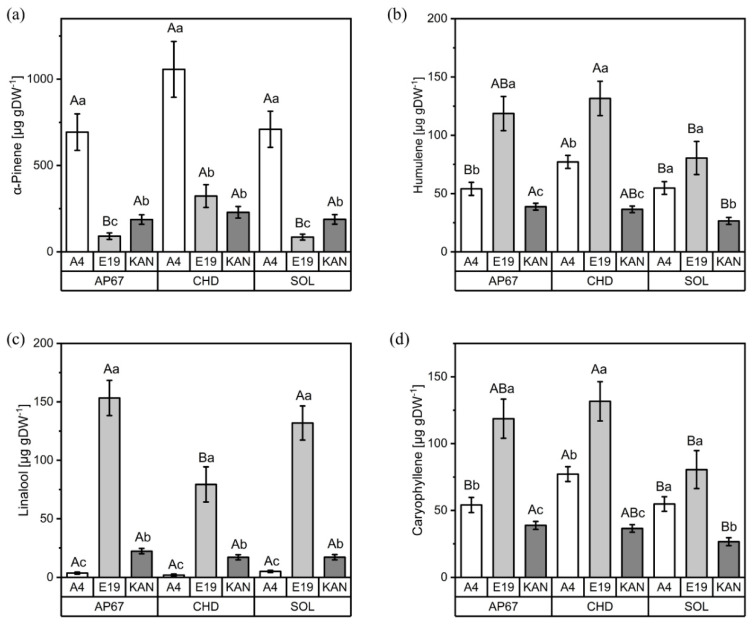
Concentrations (µg g DW^−1^) of α-pinene (**a**), humulene (**b**), linalool (**c**) and caryophyllene (**d**) for the different strains (A4, E19 and KAN) under the three light spectra (SOL, AP67 and CHD). Means covered by at least one identical letter did not differ significantly, as indicated by a Tukey test (α = 0.05). Lowercase letters indicate comparisons between strains within light spectra; uppercase letters indicate comparisons between light spectra within strains. Error bars indicate the standard errors of the means (*n* = 3).

Significant interactions between light spectra and strains were found for the terpenes α-pinene, humulene, caryophyllene and linalool. The strain A4 showed a considerably higher concentration of α-pinene than the other two strains under all light spectra (Figure 5a). Comparing light spectra, concentrations of α-pinene increased significantly under CHD (323.02 µg g DW^−1^) compared to the two LED lights, AP67 (90.85 µg g DW^−1^) and SOL (85.54 µg g DW^−1^), for the strain E19.

The highest concentration of humulene was also found under CHD (Figure 5b). Across all light spectra, humulene showed a different pattern compared to α-pinene, with the highest concentration being found for E19 (131.63 µg g DW^−1^), followed by A4 (77.14 µg g DW^−1^), whereas the highest concentration for KAN was produced under AP67 (38.76 µg g DW^−1^). The same distribution as for humulene was also observed for caryophyllene (Figure 5d).

The terpene linalool (Figure 5c) showed more pronounced differences between strains and light spectra (Figure 5c), as strain E19 had considerably higher concentrations than KAN and A4. AP67 (153.30 µg g DW^−1^) and SOL (131.89 µg g DW^−1^) increased the concentration of linalool for E19 significantly compared to CHD (79.34 µg g DW^−1^), whereas no difference between light spectra was found for A4 and KAN.

#### 3.2.2. Interactions between Light Spectra and Flower Positions

**Figure 6:**
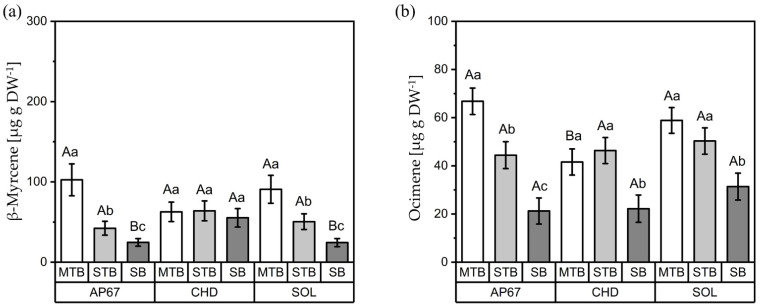
Concentrations (µg g DW ^-1^) of β-myrcene (**a**) and ocimene (**b**) for the different flower positions (MTB: main top bud, SBs: side buds, STBs: side top buds) under the three light spectra (SOL, AP67 and CHD). Means covered by at least one identical letter did not differ significantly, as indicated by a Tukey test (α = 0.05). Lowercase letters indicate comparisons between flower positions within light spectra; uppercase letters indicate comparisons between light spectra within flower positions. Error bars indicate the standard errors of the means (*n* = 3).

For the terpenes β-myrcene and ocimene, significant interactions between light spectra and flower positions were found (Figure 6). The concentration of β-myrcene showed the same trend across flower positions under AP67 and SOL in the order MTBs > STBs > SBs, with the highest concentrations in the MTBs (102.57 µg g DW^−1^) under AP67 as well as the MTBs (90.78 µg g DW^−1^) under SOL (Figure 6a). In contrast, CHD displayed a homogeneous distribution across all flower positions. Ocimene (Figure 6b) revealed a more differentiated distribution, with significantly higher concentrations in the MTBs of AP67 (66.79 µg g DW^−1^) and SOL (58.84 µg g DW^−1^). Additionally, only AP67 showed a significant concentration gradient for the MTBs, STBs and SBs. Among CHD and SOL, in contrast, only a significantly low concentration was found in the SBs.

#### 3.2.3. Interactions between Strains and Flower Positions

**Figure 7:**
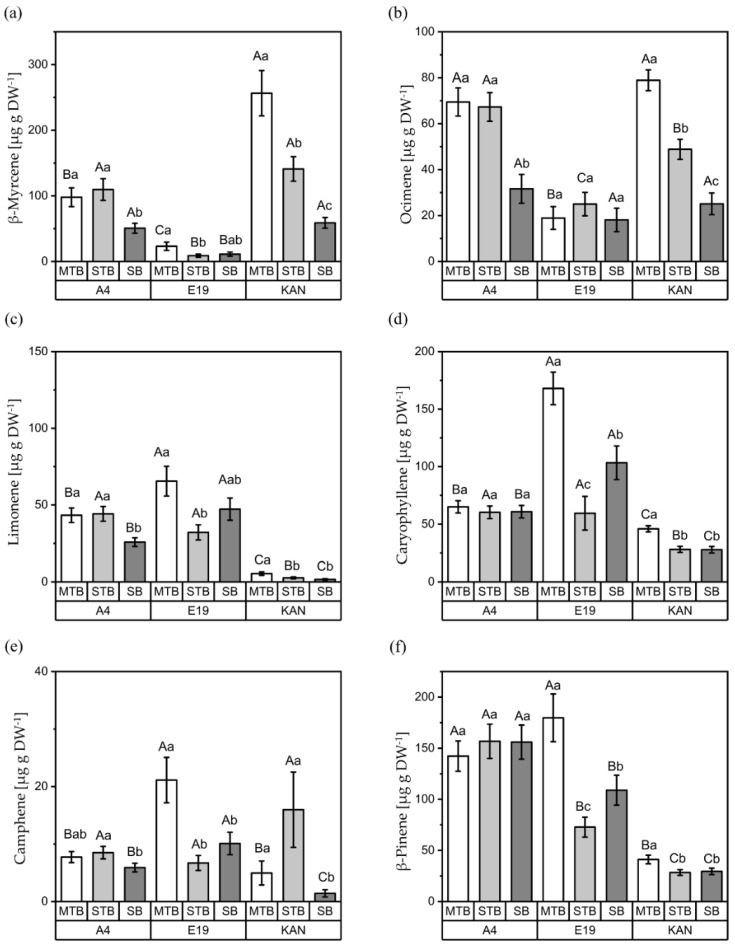
Concentrations (µg g DW^-1^) of β-myrcene (**a**), ocimene (**b**), limonene (**c**), caryophyllene (**d**), camphene (**e**) and β-pinene (**f**) for the different flower positions (MTB: main top bud, SBs: side buds, STBs: side top buds) of the three strains (A4, E19 and KAN). Means covered by at least one identical letter did not differ significantly, as indicated by a Tukey test (α = 0.05). Lowercase letters indicate comparisons between flower positions within strains; uppercase letters indicate comparisons between strains within flower positions. Error bars indicate the standard errors of the means (*n* = 3).

The interactions between strains and flower positions revealed significant differences for the terpenes β-myrcene, ocimene, limonene, caryophyllene, camphene and β-pinene (Figure 7).

A similar trend for β-myrcene and ocimene was found across flower positions for A4 and KAN. KAN displayed the highest concentration of β-myrcene, with 256.40 µg g DW^−1^ in the MTBs, which differed significantly compared to the STBs (140.99 µg g DW^−1^) and SBs (58.92 µg g DW^−1^) (Figure 7a). In contrast, in A4, both the MTBs (97.85) and STBs (109.64) exhibited nearly the same concentrations, with significant reductions in the SBs. Comparing strains, KAN showed a significantly higher concentration of β-myrcene than A4, followed by E19. The same significant concentration gradient in the flower positions was also evident for KAN regarding ocimene, with the MTBs containing a 61% higher concentration than the STBs. A4 showed the highest concentration in the MTBs and STBs, with the MTBs producing 220% more than the SBs. No significant differences could be found between the flower positions at E19.

In the case of limonene, however, E19 (65.49 µg g DW^−1^) produced significantly higher amounts than A4 (43.31 µg g DW^−1^), while KAN (5.38 µg g DW^−1^) had the lowest concentrations (Figure 7c). The strain A4 exhibited the same trend across flower positions as β-myrcene and ocimene, with similar concentrations in the MTBs (69.44 µg g DW^−1^) and STBs (67.27 µg g DW^−1^), which were significantly higher than in the SBs (31.64 µg g DW^−1^). A different distribution was found for E19, with the highest concentration in the MTBs (65.49 µg g DW^−1^), which was significantly different from that for the STBs (32.13 µg g DW^−1^).

The concentration of caryophyllene was around 64.97 µg g DW^−1^, equally distributed across all flower positions, for A4 (Figure 7d). On the other hand, E19 had a significantly heterogeneous allocation in the order MTBs (168.01 µg g DW^−1^) > SBs (103.32 µg g DW^−1^) > STBs (59.46 µg g DW^−1^). The strain KAN displayed the same pattern as for limonene, with a significantly higher concentration in the MTBs (45.92 µg g DW^−1^) compared to the other flower positions.

The concentrations of camphene displayed a very similar pattern across flower positions to caryophyllene for the strains A4 and E19 (Figure 6e). Still, the STBs of A4 were revealed to have significantly more camphene (8.50 µg g DW^−1^) than the SBs (5.89 µg g DW^−1^). As for caryophyllene, E19 had a significantly more heterogeneous distribution in the order MTBs (21.11 µg g DW^−1^) > SBs (10.09 µg g DW^−1^) > STBs (6.70 µg g DW^−1^). A distinct camphene-specific pattern was found for KAN, with significant differences in the order STBs (15.96 µg g DW^−1^) >MTBs (4.95 µg g DW^−1^) > SBs (1.42 µg g DW^−1^).

β-pinene, as well, had a similar distribution across all flower positions comparable to caryophyllene. In A4, the flower positions showed no significant differences (150 µg g DW^−1^), whereas E19 revealed concentrations in the order MTBs (179.71 µg g DW^−1^) > SBs (108.93 µg g DW^−1^) > STBs (72.78 µg g DW^−1^). For KAN, an increase of 39% was revealed in the MTBs compared to the SBs and STBs.

#### 3.2.4. Principal Component Analysis

**Figure 8:**
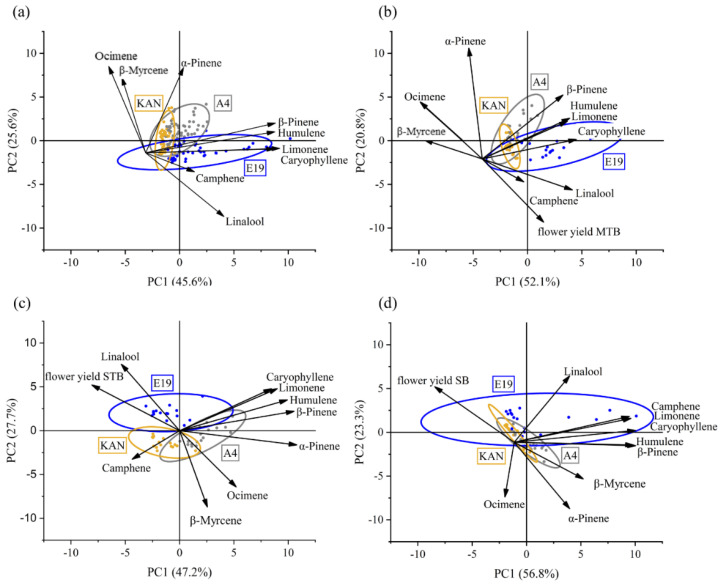
Biplot of the impact of terpene-by-strain period means on (**a**) terpene concentrations for three strains (A4, E19 and KAN), (**b**) terpene concentrations and yield of the main top buds (MTB´s) for three strains, (**c**) terpene concentrations and yield of the side top buds (STB´s) for three strains, and (**d**) terpene concentrations and yield of the side buds (SB´s) for three strains. PC1 and PC2 are principal components 1 and 2.

The biplots of the terpenes as well as their interactions with the yields for the respective flower positions showed that more than 70% of the variation can be displayed by two examined components (Figure 8).

The PCA revealed that linalool and camphene were positively correlated (Figure 8a). In addition, there was a slightly positive correlation between camphene and linalool. On the other hand, a negative correlation was detected for these two terpenes with ocimene and β-myrcene. Furthermore, a positive correlation was found between β-pinene, humulene, limonene and caryophyllene. Each strain displayed its typical terpene profile. A4 was dominated by α-pinene and β-pinene, whereas E19 accumulated more linalool, humulene and caryophyllene. On the other hand, KAN was associated with β-myrcene ocimene and α-pinene.

A further PCA was performed to analyze a possible dilution effect of the terpenes with increasing flower yield [5,6]. This was carried out for each of the three flower positions. The PCA for the MTBs (Figure 8b) indicated the same interaction as the sole consideration for only the terpenes, without the influence of the specific yield (Figure 8a), i.e., no impact of MTB yield on the respective terpene profile. For the STBs, a negative interaction between flower yield and terpene concentration was found (Figure 8c), which became more pronounced for the SBs (Figure 8d). These negative correlations were found for all terpenes, except linalool.

## 4. Discussion

### 4.1. Secondary Metabolism in Cannabis sativa L.

It is already well established in the literature that different cannabis strains have particular terpene profiles, based on which they are classified into chemovars [53,54,55]. The core terpenes present in nearly all cannabis strains are the monoterpenes β-myrcene, α-pinene and limonene, and the sesquiterpenes caryophyllene and α-humulene [3]. This is in line with the terpene profile we obtained for the strains in our study, although their concentrations varied significantly from strain to strain, especially those for limonene (Figure 7). Furthermore, our data indicated positive and negative correlations between the different terpenes (Figure 8). The positive correlation between caryophyllene and humulene is consistent with [12,53], characterizing the TPS for sesquiterpene, while indicating that the same *Cannabis sativa* L. (Cs) TPS9 gene controls the synthesis of both terpenes, which is line with the results of [53,56]. The synthesis of α-pinene is controlled by CsTPS2 [3], which most probably was present only in the tested strain A4 (Figure 5), as this strain exhibited a significantly higher α-pinene level. CsTPS35, responsible for linalool/nerolidol synthesis is, according to [4], not connected with the α-pinene synthase, which is in line with our PCA results. However, we assume a negative correlation between CsTPS35 (linalool/nerolidol) with the CsTPS15CT of β-myrcene. In addition, CsTPS9 (caryophyllene and humulene) appear to correlate positively with the CsTPSs of β-pinene and limonene (CsTPS14CT) (Figure 8). This partially confirms our hypothesis that different terpenes are positively interrelated based on shared TPS groups. Furthermore, our results indicated that, in some strains, the presence of TPS-g may trigger a down-regulation of TPS-b. Evidence that different terpenes are negatively and positively correlated has already been presented for *Cannabis sativa* L. [53,56] and mango [57]. Furthermore, the authors proved that mango strains differ due to the up- or down-regulation of the MEP and MEV pathways, which increases or decreases, respectively, the synthesis of mono- and sesquiterpenes. Each cannabis strain is characterized by a specific aroma, which is presumably based on a genetically determined activation of CsTPSs clustered in TPS groups. These TPS groups are linked to each other, whereby concentrations of mono- and sesquiterpenes depend on the enzymatic regulation of the MEV and MEP pathways and the resultant quantities of available enzymatic precursors [25]. However, our sample size was quite small (only three strains) and more research with a larger strain number in combination with gene plotting and comparisons with other cultures via PMN [58] and GNPS [59] is necessary to arrive at a more refined conclusion regarding the formation of the terpene profile of *Cannabis sativa* L.

### 4.2. Cannabis Strains and Flowers Positions

In addition to the correlations between terpenes, this study revealed different compositions of terpenes and CBDA in the individual flower positions (Figure 7). Terpene synthesis can change due to external factors, such as light, temperature and abiotic and biotic stress. Since all other factors, except light, were comparable for all strains and significant differences between flower positions were found within one strain, morphology, with associated light dispersion, has to be taken into account for the interpretation of the data (Figure 2). The strain A4 is characterized by an elongated growth, in which the MTB and STBs especially are not shaded, in contrast to the SBs, which grow close to the main stem and are therefore shaded by the main leaves. In this perspective, the secondary metabolites should be concentrated in the STBs and MTB. This trend was evident for CBDA and even more clearly expressed for terpenes. Here, significant differences between the STBs and MTBs compared to the SBs could be seen, especially for β-myrcene, limonene and ocimene. The strain KAN showed the shortest and most compact growth habit, resulting in a strong shading of SBs. This was apparent in the CBDA concentrations, as well as in those of the terpenes β-myrcene, ocimene and camphene, which concentrations differed significantly in the order MTBs > STBs > SBs. Based on these findings, E19, whose habitus is defined by longer side shoots and long internodal distances that allow better light penetration, should have a more homogeneous distribution. E19, in particular, had a very low CBDA concentration, which did not lead to significant differences between the flower positions. Unexpectedly, there was an increase towards the SBs. This was also found for the terpenes β-myrcene, limonene, ocimene and camphene, with respect to which the MTBs and SBs showed no significant differences. This might be related to less self-shading but does not explain the lower terpene concentrations in the STBs.

The theory that light intensity can increase terpene production in *Cannabis sativa* L. is supported by [5]. The authors reported increased photosynthetic capacity (A_max_) associated with higher terpene concentrations, especially for β-myrcene, limonene and caryophyllene, in relation to one cultivar under increasing light intensity. It should be noted that only the MTBs were analyzed. Therefore, shading of the inflorescences by their own canopies associated with a low photosynthetic rate [33] could explain the differences between the MTBs, STBs and SBs in our data. This is further supported by the finding that the MEP pathway, which operates in the plastids [60] and consists of a cascade of seven enzymes [61], is regulated particularly by light intensity [22]. However, this does not explain why the monoterpene camphene behaved atypically, with a higher accumulation in the STBs of KAN. GPP sinks based on genetically determined CsTPSs could be a reason for this. At present, no publication has shed light on the internal sink and source order for GPP in cannabinoid and terpene biosynthesis in *Cannabis sativa* L. We assume that in the case of A4, the synthesis of β-myrcene, limonene and ocimene served as a strong sink based on the activation of the CsTPS genes. As the formation of GPP over the MEP pathway can be promoted by light intensity [22,23], this should lead to an increase in the illuminated MTBs and STBs. This could be observed for β-myrcene, ocimene, caryophyllene and limonene for the strain KAN (Figure 7). The same principle can be applied to cannabinoid synthesis, where the interplay between PKS and the MEP pathway also plays a central role in the formation of CBGA. However, some terpenes behaved atypically; hence, we can only partially confirm our hypothesis that the shading of flowers plays an important role in the considerable variations in the secondary metabolism of the flower organs and may account for the lower terpene and CBDA concentrations in the SBs of A4 and KAN. Understanding the accumulation of secondary compounds per flower position and strain would offer growers the possibility of further diversifying their harvests and bundling flower positions of the highest quality in so-called “top-shelf” flowers.

### 4.3. Light Affecting Yield

Artificial lightning can affect primary as well as secondary metabolism [62]. In relation to yield, one of the most important modulators is the photosynthetic rate, and a high yield is directly related to this [63]. This is consistent with our data [36], as E19, the strain with the highest maximum photosynthetic rate (A_max_), also produced the highest yield compared to KAN and A4. Considering A_max_, the plants under the respective light spectra showed a maximum photosynthetic rate in the order CHD > AP67 > SOL, with a significant decrease under SOL [36]. However, the low A_max_ under SOL did not lead to significant yield losses across the strains, and an increase in yield compared to CHD was even observed. In the mentioned study, A_max_ was measured under the light source of the gas-exchange system, which is composed of red and blue LEDs and may, therefore, not represent the photosynthesis rates of leaves under more complex spectra of the light sources used. Hence, in this study, ambient photosynthetic rates were measured for additional plants (not from this experiment) of the strain KAN under the experimental light intensity of 400 µmol m^−2^ s^−1^. The ambient rates were in line with A_max_, showing significantly higher rates under CHD and AP67 compared to SOL (Table 1). For the cultivation system of *Cannabis sativa* L., the photosynthetic performances of the respective spectra were, therefore, decisive, as a change in the lighting system was accompanied by a change in photosynthetic rate, even under the same PAR. Consequently, light intensity may have to be increased or decreased to achieve the same initial yields.

### 4.4. Yield Affecting CBDA and Terpene Concentration

Recent publications indicate that an increase in yield in *Cannabis sativa* L. is not accompanied by an increase in the concentration of secondary metabolites [5] and may even lead to a dilution effect [6].

Our results for CBDA suggest a dilution effect for SBs, because significantly higher yields under the LED lights [36] resulted in lower CBDA concentrations (Figure 3). The yields of the MTBs differed significantly in the order A4 < KAN < E19; nevertheless, no deviation from the terpene profile was determined based on our PCA (Figure 8a). Therefore, we can conclude that the MTBs represent the terpene profiles of the respective strains independently of yield. The STB yields displayed the same significant differences as the MTBs, but a distinct tendency towards more negative correlations was observed. In fact, the only positive interaction occurred between STBs and linalool, the dominant terpene of E19 (Figure 5c). In the case of SBs, the negative relationship between yield and terpene concentration was most pronounced (Figure 8d). Here, all terpenes apart from linalool were negatively correlated. It is already known that in *Arabidopsis thaliana* the photosynthetic rate can influence carbohydrate metabolism as well as the shikimate pathway [64], which can lead to a decrease in secondary metabolites. Our results indicated that light intensity influenced the relationship between flower yield and terpene concentration, given that no dilution effect for the fully illuminated MTBs was found, while a dilution effect was revealed with higher flower yields for the shaded SBs.

### 4.5. Cannabinoids and Monoterpenes Modulated by R:FR Ratio

The influence of light on monoterpenes and cannabinoids is always accompanied by up- or down-regulation of the MEP pathway, in which chloroplast isoprenoids are given a core task [47]. As PIFs are direct regulators of the light-modulated expression of the MEP pathway genes [47], the shade avoidance induced by a low R:FR ratio, leading to higher PIF activity based on a reduction in PHYB [35], could therefore be a crucial factor in the modulation of secondary metabolism. The light spectra had the following R:FR ratios: AP67: 4.04, CHD: 2.83, SOL: 13.49. Based on our theory, a high R:FR ratio should result in lower PIF activity. In addition, Pfr, induced by red light, should further reduce their expression and promote the MEP pathway [47]. Therefore, the expected terpene yields in the main top buds were in the order SOL > AP67 > CHD. However, the data indicate that different terpenes respond differently to light quality. Higher β-myrcene, ocimene and linalool concentrations were found under both LED spectra compared to CHD. In contrast, the concentration of α-pinene was higher under CHD in A4 and E19. Considering all of the findings, we cannot confirm our hypothesis that a high R:FR ratio promotes monoterpene synthesis. However, our data indicated that this may be the case for CBDA.

The CBDA concentrations measured in the main top buds were highest under the SOL treatment (Figure 3). No difference could be found between A4 and KAN under the LED lights, but A4 showed significantly less CBDA under CHD. As two synthesis pathways are involved in cannabinoid synthesis, MEP and PKS, it is difficult to draw spectra-related conclusions about PKS in particular. Therefore, the hypothesis that a high R:FR ratio enhances cannabinoid or terpene concentrations remains unconfirmed.

### 4.6. Light Research on Cannabis sativa L.

Currently, apart from [42], no publication has given insights into terpene synthesis for *Cannabis sativa* L. under different light spectra. However, in the study of [42], spectra at flowering induction were changed and different PARs were applied under each treatment, making an interpretation almost impossible; in addition, there was no statistical evaluation for the single terpenes. The most recent *Cannabis sativa* L. publications focus mainly on cannabinoids, using different methodological approaches and showing widely differing results [39,65,66]. For example, [67] showed a strain-specific interaction for cannabinoids in three different strains, each under four different light sources, and it was not possible to make a clear statement on the influence of light. This is mainly due to the fact that, while cannabinoid biosynthesis is connected to the MEP pathways via GDD, influences on the cannabis-specific PKS pathway have not been investigated yet. Furthermore, spectral effects, such as those of the MEP pathway on monoterpenes, are difficult to transfer from other well-researched species, e.g., *Arabidopsis thaliana*. At present, we can only make assumptions about the influence of light on *Cannabis sativa* L. Our work, therefore, represents a crucial step in demonstrating the heterogeneous distribution of secondary metabolites under three light spectra across the plants and points to future research questions.

## 5. Conclusions

We assume that CsTPSs play a central role in the modulation of sesqui- and monoterpenes in *Cannabis sativa* L. Furthermore, there seems to be a correlation between different TPS synthase groups which are responsible for the chemovar classifications. In addition to the influence of the tested strains, we revealed different terpenes as well as different concentrations of terpenes and CBDA levels in the respective flower positions, for which we assumed canopy shading, with an associated lower photosynthetic rate, to be the cause. This encourages various pruning and defoliation techniques to enhance light-use efficiency, modify plant morphology and expose flower organs more fully to higher illumination levels. The photosynthetic rate seems to be a central element of the MEP pathway; thus, research on the photosynthetic rate in *Cannabis sativa* L. is of central importance. Regarding possible spectral effects, experiments on the effects of R:FR ratios and monochromatic light on cannabinoids and terpenes should help to gradually determine spectral influences. One of the most crucial tasks, however, in the future of *Cannabis sativa* L. research will be the genetic decoding of the internal regulation of the different pathways involved in secondary metabolism.

## Figures and Tables

**Table 1:** Photosynthetic rate (µmol CO_2_ m^−2^ s^−1^) of the strain KAN under the different light spectra (SOL, AP67 and CHD) as well as different R:FR ratios. Results are presented as mean values ± standard errors (means ± SEs). Means followed by at least one identical lower-case letter did not differ significantly at α = 0.05, within a strain or flower, as indicated by a Tukey test.

Trait	Light Spectra	PPFD	PFD_Red (µmol m^−2^ s^−1^)	PFD_Far-Red	Photosynthetic Rate (µmol CO_2_ m^−2^ s^−1^)
A light spectra	AP67	400	250	70	10.23 ± 0.33 ^a^
	CHD	400	179	82	10.44 ± 0.33 ^a^
	SOL	400	181	20	7.37 ± 0.33 ^b^
A R:FR	R	400	400	0	7.44 ± 0.09 ^b^
	R:FR 2.8	400	400	140	8.22 ± 0.09 ^a^
	R:FR 4	400	400	100	8.37 ± 0.09 ^a^
	R:FR 13.5	400	400	30	7.64 ± 0.09 ^b^
*p*-values, light spectra					
					0.0049
*p*-values, R:FR					
					0.0011

## Data Availability

Data sharing is not applicable to this article, as all new data are contained within the article.

## References

[1] Aliferis K.A., Bernard-Perron D. (2020). Cannabinomics: Application of Metabolomics in Cannabis (*Cannabis sativa* L.) Research and Development. Front. Plant Sci..

[2] Booth J.K., Bohlmann J. (2019). Terpenes in *Cannabis sativa*—From plant genome to humans. Plant Sci..

[3] Booth J.K., Yuen M.M.S., Jancsik S., Madilao L.L., Page J.E., Bohlmann J. (2020). Terpene Synthases and Terpene Variation in *Cannabis sativa*. Plant Physiol..

[4] Zager J.J., Lange I., Srividya N., Smith A., Lange B.M. (2019). Gene Networks Underlying Cannabinoid and Terpenoid Accumulation in Cannabis. Plant Physiol..

[5] Rodriguez-Morrison V., Llewellyn D., Zheng Y. (2021). Cannabis Yield, Potency, and Leaf Photosynthesis Respond Differently to Increasing Light Levels in an Indoor Environment. Front. Plant Sci..

[6] Saloner A., Bernstein N. (2021). Nitrogen supply affects cannabinoid and terpenoid profile in medical cannabis (*Cannabis sativa* L.). Ind. Crop. Prod..

[7] Saloner A., Bernstein N. (2020). Response of Medical Cannabis (*Cannabis sativa* L.) to Nitrogen Supply Under Long Photoperiod. Front. Plant Sci..

[8] Danziger N., Bernstein N. (2021). Plant architecture manipulation increases cannabinoid standardization in ‘drug-type’ medical cannabis. Ind. Crop. Prod..

[9] Crispim Massuela D., Hartung J., Munz S., Erpenbach F., Graeff-Hönninger S. (2022). Impact of Harvest Time and Pruning Technique on Total CBD Concentration and Yield of Medicinal Cannabis. Plants.

[10] Gagne S.J., Stout J.M., Liu E., Boubakir Z., Clark S.M., Page J.E. (2012). Identification of olivetolic acid cyclase from *Cannabis sativa* reveals a unique catalytic route to plant polyketides. Proc. Natl. Acad. Sci. USA.

[11] Jin D., Dai K., Xie Z., Chen J. (2020). Secondary Metabolites Profiled in Cannabis Inflorescences, Leaves, Stem Barks, and Roots for Medicinal Purposes. Sci. Rep..

[12] Booth J.K., Page J.E., Bohlmann J. (2017). Terpene synthases from *Cannabis sativa*. PLoS ONE.

[13] Bouvier F., Rahier A., Camara B. (2005). Biogenesis, molecular regulation and function of plant isoprenoids. Prog. Lipid Res..

[14] Andre C.M., Hausman J.-F., Guerriero G. (2016). *Cannabis sativa*: The Plant of the Thousand and One Molecules. Front. Plant Sci..

[15] Flores-Sanchez I.J., Verpoorte R. (2008). Secondary metabolism in cannabis. Phytochem. Rev..

[16] Fellermeier M., Zenk M.H. (1998). Prenylation of olivetolate by a hemp transferase yields cannabigerolic acid, the precursor of tetrahydrocannabinol. FEBS Lett..

[17] Luo X., Reiter M.A., d’Espaux L., Wong J., Denby C.M., Lechner A., Zhang Y., Grzybowski A.T., Harth S., Lin W. (2019). Complete biosynthesis of cannabinoids and their unnatural analogues in yeast. Nature.

[18] Vranová E., Coman D., Gruissem W. (2013). Network analysis of the MVA and MEP pathways for isoprenoid synthesis. Annu. Rev. Plant Biol..

[19] Ghassemian M., Lutes J., Tepperman J.M., Chang H.-S., Zhu T., Wang X., Quail P.H., Lange B.M. (2006). Integrative analysis of transcript and metabolite profiling data sets to evaluate the regulation of biochemical pathways during photomorphogenesis. Arch. Biochem. Biophys..

[20] Soto G., Stritzler M., Lisi C., Alleva K., Pagano M.E., Ardila F., Mozzicafreddo M., Cuccioloni M., Angeletti M., Ayub N.D. (2011). Acetoacetyl-CoA thiolase regulates the mevalonate pathway during abiotic stress adaptation. J. Exp. Bot..

[21] Rodriguez-Concepcion, Gruissem (1999). Arachidonic acid alters tomato HMG expression and fruit growth and induces 3-hydroxy-3-methylglutaryl coenzyme A reductase-independent lycopene accumulation. Plant Physiol..

[22] Mongélard G., Seemann M., Boisson A.-M., Rohmer M., Bligny R., Rivasseau C. (2011). Measurement of carbon flux through the MEP pathway for isoprenoid synthesis by (31)P-NMR spectroscopy after specific inhibition of 2-C-methyl-d-erythritol 2,4-cyclodiphosphate reductase. Effect of light and temperature. Plant Cell Environ..

[23] Srinath M., Shailaja A., Bindu B.B.V., Giri C.C. (2022). Comparative analysis of biomass, ethrel elicitation, light induced differential MVA/MEP pathway gene expression and andrographolide production in adventitious root cultures of *Andrographis paniculata* (Burm. F.) Nees. Plant Cell Tissue Organ Cult..

[24] Desaulniers Brousseau V., Wu B.-S., MacPherson S., Morello V., Lefsrud M. (2021). Cannabinoids and Terpenes: How Production of Photo-Protectants Can Be Manipulated to Enhance *Cannabis sativa* L. Phytochemistry. Front. Plant Sci..

[25] Tholl D. (2015). Biosynthesis and biological functions of terpenoids in plants. Adv. Biochem. Eng. Biotechnol..

[26] Chen F., Tholl D., Bohlmann J., Pichersky E. (2011). The family of terpene synthases in plants: A mid-size family of genes for specialized metabolism that is highly diversified throughout the kingdom. Plant J..

[27] Karunanithi P.S., Zerbe P. (2019). Terpene Synthases as Metabolic Gatekeepers in the Evolution of Plant Terpenoid Chemical Diversity. Front. Plant Sci..

[28] Michael R., Ranjan A., Kumar R.S., Pathak P.K., Trivedi P.K. (2020). Light-regulated expression of terpene synthase gene, AtTPS03, is controlled by the bZIP transcription factor, HY5, in Arabidopsis thaliana. Biochem. Biophys. Res. Commun..

[29] Kawoosa T., Singh H., Kumar A., Sharma S.K., Devi K., Dutt S., Vats S.K., Sharma M., Ahuja P.S., Kumar S. (2010). Light and temperature regulated terpene biosynthesis: Hepatoprotective monoterpene picroside accumulation in *Picrorhiza kurrooa*. Funct Integr. Genom..

[30] Klepzig K.D., Kruger E.L., Smalley E.B., Raffa K.F. (1995). Effects of biotic and abiotic stress on induced accumulation of terpenes and phenolics in red pines inoculated with bark beetle-vectored fungus. J. Chem. Ecol..

[31] Aizpurua-Olaizola O., Soydaner U., Öztürk E., Schibano D., Simsir Y., Navarro P., Etxebarria N., Usobiaga A. (2016). Evolution of the Cannabinoid and Terpene Content during the Growth of *Cannabis sativa* Plants from Different Chemotypes. J. Nat. Prod..

[32] Knudsen J.T., Eriksson R., Gershenzon J., Ståhl B. (2006). Diversity and Distribution of Floral Scent. Bot. Rev..

[33] Figueiredo A.C., Barroso J.G., Pedro L.G., Scheffer J.J.C. (2008). Factors affecting secondary metabolite production in plants: Volatile components and essential oils. Flavour Fragr. J..

[34] Kleine S., Müller C. (2011). Intraspecific plant chemical diversity and its relation to herbivory. Oecologia.

[35] Casal J.J. (2013). Photoreceptor Signaling Networks in Plant Responses to Shade. Annu. Rev. Plant Biol..

[36] Reichel P., Munz S., Hartung J., Präger A., Kotiranta S., Burgel L., Schober T., Graeff-Hönninger S. (2021). Impact of Three Different Light Spectra on the Yield, Morphology and Growth Trajectory of Three Different *Cannabis sativa* L. Strains. Plants.

[37] Eichhorn Bilodeau S., Wu B.-S., Rufyikiri A.-S., MacPherson S., Lefsrud M. (2019). An Update on Plant Photobiology and Implications for Cannabis Production. Front. Plant Sci..

[38] McCree K.J. (1971). The action spectrum, absorptance and quantum yield of photosynthesis in crop plants. Agric. Meteorol..

[39] Magagnini G., Grassi G., Kotiranta S. (2018). The Effect of Light Spectrum on the Morphology and Cannabinoid Content of *Cannabis sativa* L. Med. Cannabis Cannaboids.

[40] Westmoreland F.M., Kusuma P., Bugbee B. (2021). Cannabis lighting: Decreasing blue photon fraction increases yield but efficacy is more important for cost effective production of cannabinoids. PLoS ONE.

[41] Hawley D., Graham T., Stasiak M., Dixon M. (2018). Improving Cannabis Bud Quality and Yield with Subcanopy Lighting. HortScience.

[42] Namdar D., Charuvi D., Ajjampura V., Mazuz M., Ion A., Kamara I., Koltai H. (2019). LED lighting affects the composition and biological activity of *Cannabis sativa* secondary metabolites. Ind. Crops Prod..

[43] Shinomura T., Uchida K., Furuya M. (2000). Elementary processes of photoperception by phytochrome A for high-irradiance response of hypocotyl elongation in Arabidopsis. Plant Physiol..

[44] Mathews S. (2006). Phytochrome-mediated development in land plants: Red light sensing evolves to meet the challenges of changing light environments. Mol. Ecol..

[45] Endo M., Tanigawa Y., Murakami T., Araki T., Nagatani A. (2013). PHYTOCHROME-DEPENDENT LATE-FLOWERING accelerates flowering through physical interactions with phytochrome B and CONSTANS. Proc. Natl. Acad. Sci. USA.

[46] Llorente B., D’Andrea L., Ruiz-Sola M.A., Botterweg E., Pulido P., Andilla J., Loza-Alvarez P., Rodriguez-Concepcion M. (2016). Tomato fruit carotenoid biosynthesis is adjusted to actual ripening progression by a light-dependent mechanism. Plant J..

[47] Chenge-Espinosa M., Cordoba E., Romero-Guido C., Toledo-Ortiz G., León P. (2018). Shedding light on the methylerythritol phosphate (MEP)-pathway: Long hypocotyl 5 (HY5)/phytochrome-interacting factors (PIFs) transcription factors modulating key limiting steps. Plant J..

[48] Burgel L., Hartung J., Graeff-Hönninger S. (2020). Impact of Different Growing Substrates on Growth, Yield and Cannabinoid Content of Two *Cannabis sativa* L. Genotypes in a Pot Culture. Horticulturae.

[49] Hitz T., Graeff-Hönninger S., Munz S. (2020). Modelling of Soybean (*Glycine max* (L.) Merr.) Response to Blue Light Intensity in Controlled Environments. Plants.

[50] Honnold R., Kubas R., Macherone A. (2017). Analysis of Terpenes in Cannabis Using the Agilent 7697A/7890B/5977B Headspace GC-MSD System.

[51] Burgel L., Hartung J., Schibano D., Graeff-Hönninger S. (2020). Impact of Different Phytohormones on Morphology, Yield and Cannabinoid Content of *Cannabis sativa* L. Plants.

[52] Piepho H.P., Buchse A., Emrich K. (2003). A Hitchhiker’s Guide to Mixed Models for Randomized Experiments. J. Agron. Crop. Sci..

[53] Hazekamp A., Fischedick J.T. (2012). Cannabis—From cultivar to chemovar. Drug Test. Anal..

[54] Hazekamp A., Tejkalová K., Papadimitriou S. (2016). Cannabis: From Cultivar to Chemovar II—A Metabolomics Approach to Cannabis Classification. Cannabis Cannabinoid Res..

[55] Reimann-Philipp U., Speck M., Orser C., Johnson S., Hilyard A., Turner H., Stokes A.J., Small-Howard A.L. (2020). Cannabis Chemovar Nomenclature Misrepresents Chemical and Genetic Diversity; Survey of Variations in Chemical Profiles and Genetic Markers in Nevada Medical Cannabis Samples. Cannabis Cannabinoid Res..

[56] Elzinga S., Fischedick J., Podkolinski R., Raber J.C. (2015). Cannabinoids and Terpenes as Chemotaxonomic Markers in Cannabis. Nat. Prod. Chem. Res..

[57] Suh J.H., Madden R.T., Sung J., Chambers A.H., Crane J., Wang Y. (2021). Pathway-Based Metabolomics Analysis Reveals Biosynthesis of Key Flavor Compounds in Mango. J. Agric. Food Chem..

[58] Hawkins C., Ginzburg D., Zhao K., Dwyer W., Xue B., Xu A., Rice S., Cole B., Paley S., Karp P. (2021). Plant Metabolic Network 15: A resource of genome-wide metabolism databases for 126 plants and algae. J. Integr. Plant Biol..

[59] Wang M., Carver J.J., Phelan V.V., Sanchez L.M., Garg N., Peng Y., Nguyen D.D., Watrous J., Kapono C.A., Luzzatto-Knaan T. (2016). Sharing and community curation of mass spectrometry data with Global Natural Products Social Molecular Networking. Nat. Biotechnol..

[60] Rodríguez-Concepción M., Boronat A. (2015). Breaking new ground in the regulation of the early steps of plant isoprenoid biosynthesis. Curr. Opin. Plant Biol..

[61] Phillips M.A., León P., Boronat A., Rodríguez-Concepción M. (2008). The plastidial MEP pathway: Unified nomenclature and resources. Trends Plant Sci..

[62] Darko E., Heydarizadeh P., Schoefs B., Sabzalian M.R. (2014). Photosynthesis under artificial light: The shift in primary and secondary metabolism. Philos. Trans. R. Soc. B Biol. Sci..

[63] Long S.P., Zhu X.-G., Naidu S.L., Ort D.R. (2006). Can improvement in photosynthesis increase crop yields?. Plant Cell Environ..

[64] Janacek S.H., Trenkamp S., Palmer B., Brown N.J., Parsley K., Stanley S., Astley H.M., Rolfe S.A., Paul Quick W., Fernie A.R. (2009). Photosynthesis in cells around veins of the C(3) plant Arabidopsis thaliana is important for both the shikimate pathway and leaf senescence as well as contributing to plant fitness. Plant J..

[65] Wei X., Zhao X., Long S., Xiao Q., Guo Y., Qiu C., Qiu H., Wang Y. (2021). Wavelengths of LED light affect the growth and cannabidiol content in *Cannabis sativa* L. Ind. Crops Prod..

[66] Islam M.J., Ryu B.R., Azad M.O.K., Rahman M.H., Cheong E.J., Lim J.-D., Lim Y.-S. (2021). Cannabinoids Accumulation in Hemp (*Cannabis sativa* L.) Plants under LED Light Spectra and Their Discrete Role as a Stress Marker. Biology.

[67] Danziger N., Bernstein N. (2021). Light matters: Effect of light spectra on cannabinoid profile and plant development of medical cannabis (*Cannabis sativa* L.). Ind. Crops Prod..

